# Systematic reviews for policy-making – critical reflections are needed

**DOI:** 10.1186/s12961-018-0387-9

**Published:** 2018-11-20

**Authors:** Kirsti Malterud, Anne Karen Bjelland, Kari Tove Elvbakken

**Affiliations:** 1 Research Unit for General Practice, NORCE Norwegian Research Centre, Bergen, Norway; 2 Department of Global Public Health and Primary Care, University of Bergen, Bergen, Norway; 3 The Research Unit for General Practice and Section of General Practice, Department of Public Health, University of Copenhagen, Copenhagen, Denmark; 4 Department of Social Anthropology, University of Bergen, Bergen, Norway; 5 Department of Administration and Organization Theory, University of Bergen, Bergen, Norway

In 2016, we wrote an article discussing the relationship between evidenced-based medicine (EBM) and evidence-based policy, informed by the particular evidence supposed to constitute the basis for decision-making [1]. Systematic reviews (SRs) with meta-analyses were elaborated within the EBM tradition as a tool for the development of evidence by synthesis and assessment of research findings [2]. The article presents a case study of SRs from the Norwegian Knowledge Centre for the Health Services (NOKC), where Atle Fretheim holds a leadership position. In a Letter to the Editor [3], Fretheim has criticised our methodological approach. Below, we provide a summary of the content of the article and respond to Fretheim’s comments.

We identified and organised the official publications from NOKC to an empirical corpus of typical ‘top-of-the-line’ evidence represented by SRs adhering to EBM standards. From a total of 151 SRs published by the NOKC from 2004 and 2013, a purposive subsample (including all 14 SRs published in 2012) was examined, addressing their potential as policy decision tools.

Supported by theoretical perspectives from rhetoric of health and medicine, we assessed and interpreted the persuasive power of the conclusions mediated by the concepts used, taking terms indicating positions of certainty or reluctance as our point of departure [4]. Analogous to policy-makers’ perception of such concepts, we deliberately took up a lay subject position interpreting these terms in everyday language. We did not explore the impact of the conclusions from reports for specific policy decisions.

In 2012, 57,368 studies were screened, identifying 351 that were included for synthesis in 14 SRs. For each SR, the average number of hits was 4098 (range 263–10,188) and on average 25 (range 3–91) studies were included. Caution in various forms was advocated as the major rhetorical pattern in the SR conclusions. Some of the SRs stated that no certain conclusions could be drawn, while others used very cautious terminology in their conclusions, such as ‘probably increases’, ‘possibly increases’, ‘increases perhaps’, ‘may reduce’, ‘uncertain’, or ‘difficult to conclude’. These reservations were apparently reflecting the GRADE rating system [5]. For one SR alone, the grading concluded that documentation was extensive and high-quality, whereas another reported that substantial documentation allowed some conclusions. Conclusions in the remaining 12 SRs were characterised by overarching caution in every case.

Rhetorical analysis is an interpretative methodology exploring interaction between arguments, actors and policy. Assessment of how a statement may function as an argument can be conducted in different ways, depending on purpose and context. We read Fretheim’s comments as a fundamental disagreement upon paradigms. Highly skilled in statistical meta-analysis, Fretheim dismisses the assumption of subjective judgements and theoretical reflection as essential elements of interpretative research methods. Assessing validity by trying to replicate analysis and expecting identical findings makes no sense, since there is no single correct answer to the questions asked in this kind of studies. This does not mean that the results are casual outcomes of cherry picking, but rather that different preconceptions, theoretical frameworks, background, positioning and methodological approaches have an impact on the interpretations and explain different findings. Referring to Segal [4], we declared our strategy for interpretation, as specified in our article: “*…we assessed the persuasive power of the conclusions mediated by the language used, especially with regard to terms indicating positions of certainty or reluctance*” [1].

In Fretheim’s judgement, 4 of the 14 reports included findings without major reservations about uncertainty. Our interpretation – with a different aim, a different method for analysis and a different philosophical foundation – implied that the conclusions of 12 of the reports were characterised by major caution regarding their potential as policy decision tools. We do not argue that Fretheim’s analysis is wrong. In fact, Fretheim’s judgement supports our suggestion that most of the reports advised major caution. The minor discrepancies between our assessments of the reports are easily explained by different interpretation strategies. Given Fretheim’s methodological position, it is no surprise that his understanding of the conceptual validity of the cautious terminology is dissimilar from ours. His institution has been pivotal in the development of the GRADE system, which offers a tool for systematic assessment of the evidence quality and strength of recommendations [5]. Fretheim’s arguments indicate that his interpretation of the adjectives used in the conclusions of the articles corresponds with the standards represented by this methodological framework [3]. Our point of departure, on the other hand, was to explore the clarity of advice for decision-making mediated by the language used in the conclusions. Our analysis did not take the connotation of the GRADE terminology for granted but examined the statements as the basis for decision-making.

Although we argued in the article that the SR methodology is better suited for synthesis of medication efficacy studies than for complex public health interventions, we did not assume or claim that any SR about medication efficacy studies would serve as an adequate policy decision tool. Discussing the potential of SRs concerning medication efficacy studies, we believe that Fretheim’s expression “*excluded*” [3] refers to our negative assessment of such a study (which actually seems to concur with his own).

Writing this article, we intended to offer critical reflection upon the tools used for development of evidence. Although our analysis has demonstrated some limitations for SRs, we do not argue that they have no place in policy-making processes in general. However, several of the SRs in our sample dealt with the synthesis of randomised controlled trials from complex and contextually dependent interventions, which are neither easily conducted nor standardised [6, 7]. We agree with Fretheim that SRs where high quality evidence and documentation have not been identified may also provide relevant information. However, our analysis demonstrated that it was not just a minority of SRs from our sample presenting conclusions of limited utility for decision-making. We may therefore ask whether the use of SRs was really an adequate strategy for the delivery of evidence in many of these cases. If the question to be answered and the methodology to provide the answer are not sufficiently compatible, it would be better to reject that specific commission or to develop other kinds of evidence, rather than forcing the question into confined SR frames. Finally, we share Fretheim’s concern that critical appraisal of synthesised evidence should be conducted to ensure that the documentation holds sufficiently high quality. The GRADE criteria may possibly have been set too strictly. An alternative explanation is that we have interpreted the GRADE terminology expressions in the conclusions more literally than intended by the NOKC – as may also be done by policy-makers.

It is possible to dismiss the contemporary wave of science scepticism without believing that research knowledge is a universal resolution to any problem. The ‘knowledge translation’ metaphor represents the ‘know-do’ gap to be bridged between scientific facts and policy-making as a simple pipeline model [8, 9], often expected to be fed by SRs. Our study has demonstrated that EBM and the SR methodology are not necessarily suited to provide knowledge for every kind of policy decision-making.

## References

[1] Malterud K, Bjelland AK, Elvbakken KT (2016). Evidence-based medicine - an appropriate tool for evidence-based health policy? A case study from Norway. Health Res Policy Syst.

[2] Cochrane handbook for systematic reviews of interventions Version 5.1.0 [updated March 2011]. http://handbook.cochrane.org/. Accessed 6 Nov 2018.

[3] Fretheim A. Subjective judgements – no more, no less? A response to Malterud, Bjelland and Elvbakken. Health Res Policy Syst. 2018;16. 10.1186/s12961-018-0386-x PMC6245815 30458794

[4] Segal JZ (2008). Health and the Rhetoric of Medicine.

[5] Guyatt G, Oxman AD, Vist GE, Kunz R, Falck-Ytter Y, Alonso-Coello P, Schunemann HJ (2008). GRADE: an emerging consensus on rating quality of evidence and strength of recommendations. BMJ.

[6] Dobrow MJ, Goel V, Upshur RE (2004). Evidence-based health policy: context and utilisation. Soc Sci Med.

[7] Greenhalgh T, Malterud K (2017). Systematic reviews for policymaking: muddling through. Am J Public Health.

[8] Oliver K, Lorenc T, Innvaer S (2014). New directions in evidence-based policy research: a critical analysis of the literature. Health Res Policy Syst.

[9] Greenhalgh T, Wieringa S (2011). Is it time to drop the ‘knowledge translation’ metaphor? A critical literature review. J R Soc Med.

